# A Review of HER4 (ErbB4) Kinase, Its Impact on Cancer, and Its Inhibitors

**DOI:** 10.3390/molecules26237376

**Published:** 2021-12-05

**Authors:** Mohammed I. El-Gamal, Nada H. Mewafi, Nada E. Abdelmotteleb, Minnatullah A. Emara, Hamadeh Tarazi, Rawan M. Sbenati, Moustafa M. Madkour, Seyed-Omar Zaraei, Afnan I. Shahin, Hanan S. Anbar

**Affiliations:** 1 College of Pharmacy, University of Sharjah, Sharjah 27272, United Arab Emirates; u16103115@sharjah.ac.ae (N.H.M.); u16103110@sharjah.ac.ae (N.E.A.); u16103780@sharjah.ac.ae (M.A.E.); htarazi@sharjah.ac.ae (H.T.); 2 Sharjah Institute for Medical Research, University of Sharjah, Sharjah 27272, United Arab Emirates; u19104619@sharjah.ac.ae (R.M.S.); moustafatrika22@hotmail.com (M.M.M.); holor@live.com (S.-O.Z.); u21102889@sharjah.ac.ae (A.I.S.); 3 Department of Medicinal Chemistry, Faculty of Pharmacy, Mansoura University, Mansoura 35516, Egypt; 4 Department of Clinical Pharmacy and Pharmacotherapeutics, Dubai Pharmacy College for Girls, Dubai 19099, United Arab Emirates

**Keywords:** HER4, ErbB4, cancer, kinase inhibitors, structure-activity relationship

## Abstract

HER4 is a receptor tyrosine kinase that is required for the evolution of normal body systems such as cardiovascular, nervous, and endocrine systems, especially the mammary glands. It is activated through ligand binding and activates MAPKs and PI3K/AKT pathways. HER4 is commonly expressed in many human tissues, both adult and fetal. It is important to understand the role of HER4 in the treatment of many disorders. Many studies were also conducted on the role of HER4 in tumors and its tumor suppressor function. Mostly, overexpression of HER4 kinase results in cancer development. In the present article, we reviewed the structure, location, ligands, physiological functions of HER4, and its relationship to different cancer types. HER4 inhibitors reported mainly from 2016 to the present were reviewed as well.

## 1. Introduction

**Figure 1:**
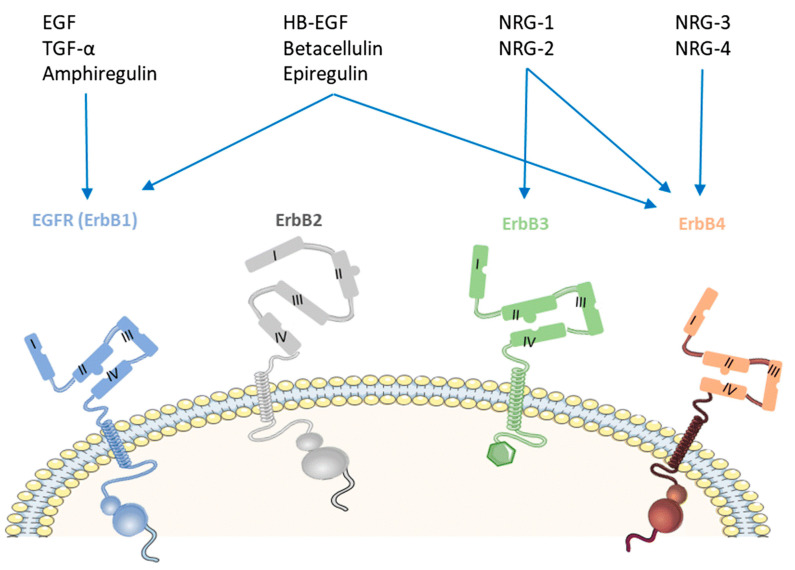
The four members of epidermal growth factor [2]. Reprinted with permission from ref. [2]. Copyright 2020 Springer Nature.

HER4, also known as ErbB4, is a member of epidermal growth factor receptor (EGFR) or ErbB family. HER4 is necessary for normal development of body systems including heart, nervous system, and mammary gland [1]. EGFR/ErbB1/HER1, ErbB2/HER2, ErbB3/HER3, and ErbB4/HER4 are members of this receptor tyrosine kinase family, as shown in Figure 1 [2]. HER4 is the only member of this family of receptors with growth inhibiting properties [2,3,4]. HER4 can activate related genes in the nucleus when combined with epidermal growth factor (EGF), promoting cell division and proliferation [5].

The binding of ligands to ErbBs causes a change in the receptor shape, which leads to dimerization either with itself to form a homodimer, or with other ErbBs to form a heterodimer [5].

When human epidermal growth factor receptors (HER) are highly expressed and activated; this often results in poor patient prognosis and advances the stages of tumor [3]. However, under normal conditions, this receptor is essential for normal development [2].

Ligand binding activates the HER4 tyrosine kinase like other ErbB receptors, which in turn activates the mitogen-activated protein kinases (MAPKs) and phosphoinositide 3-kinase (PI3K)/AKT pathways [6,7]. As a result, activation of HER4 somatic mutation is significant in a variety of human cancer [6].

In most cases, overexpression of kinases results in cancer development, therefore, many drugs are being developed by scientists to inhibit these receptors [5]. Most of the drugs that inhibit HER4 usually bind irreversibly to the ATP-binding site.

In the present article, we reviewed the structure, location, ligands, and functions of HER4 kinase, and its relationship to different types of cancer. HER4 inhibitors reported mainly from 2016 to the present were reviewed as well.

## 2. Structure of HER4 Kinase

The amino acid sequence of receptor protein-tyrosine kinase (EGFR) was first determined by Ullrich et al. using cDNA sequence analysis [8]. The glycosylated extracellular domain of the HER family is divided into four domains: ligand binding takes place in domains I and III, formation of disulfide bond takes place in domains II and IV where multiple cysteine residues are found. Moreover, hetero and homodimer formation takes place in domain II. In total, 19 to 25 amino acid residues in a single transmembrane segment and around 540 amino acid residues in an intracellular section are accompanied by the extracellular domain [9,10]

HER4 has a molar mass of 180 kD where the cytoplasmic domain shows 79% homology with the corresponding domain of EGFR and 77% homology with c-HER2 [11].

Using the homology cloning method, HER4’s cDNA sequence was identified after cloning from a human mammary carcinoma cell line [12]. The same-sized ectodomain and cytoplasmic domains are separated by a single transmembrane domain. Ectodomain is made up of a cleaved signal sequence and two cysteine-rich regions; almost all the 50 ectodomain cysteine residues of EGFR are preserved in HER4. Domain III was shown to mediate growth factor binding in comparison with EGFR. HER4 has a comparatively longer stalk region between domain IV and the transmembrane domain, which may make it uniquely susceptible to ectodomain cleavage within this receptor family [13].

All HER receptors have a cytoplasmic ambit that consists of a tyrosine kinase domain, a juxtamembrane region and a carboxy terminal domain. The residues of tyrosine autophosphorylation in EGFR that were found are all preserved in HER4. Although there are mutations in the HER3 kinase domain that considerably lessens its kinase function, these modifications were not found in HER4 kinase domain [14]. 

All protein kinases, including those of the HER family, are associated with two general forms of conformational movements. The active form of HER4 has the β4vv-lysine– αC-glutamate salt bridge (e.g., 3BCE) and the inactive form has the β4-lysine–DFG-aspartate salt bridge [15]. 

Crystallographic studies of HER kinase domain reported that the development of asymmetric dimer between a “donor” (activator) monomer’s *C*-lobe and an adjacent “acceptor” (receiver) monomer’s *N*-lobe is a typical structural mechanism necessary to attain maximum kinase activation [16,17,18,19].

**Figure 2:**
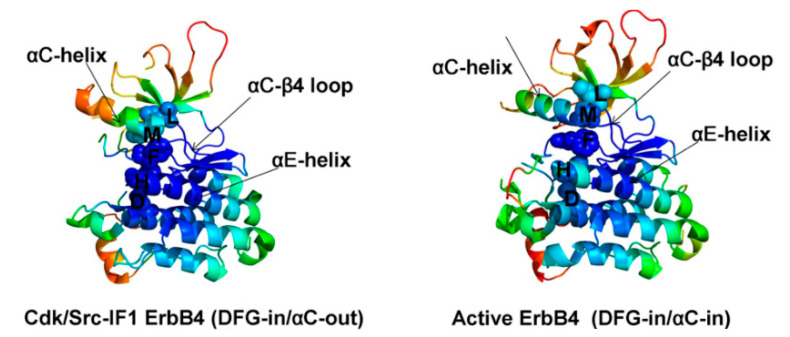
The left panel shows HER4 in Cdk/Src-IF1 form and the right panel shows the active form [20].

The local contact density obtained from structure-based network analysis is shown for the Cdk/Src-IF1 and active states of HER4 (Figure 2). The spine residues in the HER4 structures are M747, L758, H816, F837, and D877 [20].

The residues of the R spine in EGFR (M766, L777, F856, D896, H835) and HER4 (M747, H816, F837, L758, D877) are closely related, showing the structural integrity of the HRD and DFG motifs, while the residues of the αC-β4/αC-helix interface (M766, L777 in EGFR and M747, L758 in HER4) label the boundary between high and low structural stability regions [20]. 

Upon mutation of the 19th and 113th amino acid residues at the *C*-terminal, HER4 protein biological nature was not affected. However, its secondary structure was altered, and protein binding sites were nearby two mutation regions [21]. 

In addition, additional binding sites were found after mutation introduction. Tertiary modeling of the structure showed that HER4’s local structure was modified from an α-helical conformation which is the protein’s functional site to a β-chain folding structure. The mutation caused the HER4 receptor to bind to the ligand of neuregulin 1 without forming a dimer, interrupting the signal transduction pathway, and affecting the role of HER4. Expert Protein Analysis System (ExPASy) was used to analyze physiological changes in the properties after the carboxyl end mutation of the intracellular portion of HER4 [21]. 

## 3. Location of HER4 Kinase 

Localization of HER family members may be the outcome of three basic steps. In the beginning, by targeting receptor-containing vesicles to certain sites, newly synthesized receptors will be delivered to plasma membrane subdomains. To that site, they are anchored by unique intracellular and cell-surface proteins tether receptors. Protein anchoring can also regulate receptor responsiveness to stimulate growth factors or facilitate interactions between the HER family and other signaling pathways. At last, receptors are withdrawn from the cell surface and separated into intracellular compartments responsible for protein recycling or degradation. HER would be provided by cells to the sites responsible for signal transduction and sustain the receptor levels needed for biological activity [22].

Receptor tyrosine kinases are mainly located on the cell surface in limited ranges in order to maintain normal development and maintenance of tissues [22]. The HER4 gene is localized on chromosome 2q33.3–34 [23], but a distinct chromosome is assigned for each one of the HER receptor kinases family. HER4 sites for autophosphorylation are not represented but these sites are localized in the carboxy-terminal domain within other receptors in this family [14]. In mammalian skeletal muscles, HER4 is precisely located on the postsynaptic side of neuromuscular junctions in order to bind to neuregulin growth factors which are secreted by innervating neurons [22]. In addition, HER4 expression was detected in the epidermis, expressed more strongly in the basal layer compared to suprabasal layer. Expression of HER4 was confirmed using four separate antibodies with the same mode of expression in the basal layer of the epidermis [24]. Levels of expression of HER4 extracellular (JM-α and JM-β) were analyzed in invasive breast cancer patients [25]. HER4 was located mainly in the cell membrane of the intact heart [26]. The majority of HER4 cardiac myocytes are present in caveolin-enriched micro domains, most likely caveola [14]. 

## 4. Ligands of HER4 Kinase

Seven ligands are known to bind to HER4 proteins provoking conformational changes that result in its activation and signaling. These ligands are divided into two classes: the heregulins, also referred to as neuregulin (NEU) gene, and few ligands of the epidermal growth factor (EGF) family of EGFR/ErbB1. The term heregulins will be used throughout this review for uniformity purposes. Four types of heregulins are found (1, 2, 3, and 4), their recognition of HER4 is variable [26,27,28]. Several studies have determined the capacity of different heregulins in HER4 activation, particularly heregulins 3 and 4 which demonstrated their high affinity, binding properties, and their role in receptor activation [29,30]. On the other hand, heregulins 1 and 2 fail to recognize HER4 [31,32].

As for the epidermal growth factor (EGF) family of EGFR/ErbB1, few ligands have shown their agonistic potential on HER4, such as epiregulin (EPR), betacellulin (BTC) and heparin binding-EGF (HB-EGF). Epiregulin (EPR) and betacellulin (BTC) are amongst the founding members of the epidermal growth factor (EGF) family of EGFR/ErbB1 [33]. Activation of HER4 by its agonist EPR can be influenced and regulated by EGFR or HER2, due to the proposed mechanism of the receptor trans-modulation and heterodimerization. Low affinity hormone-receptor interactions are controlling the receptor’s heterodimerization and heterotypic receptor-receptor contacts, followed by the receptor kinase domains’ cross-phosphorylation [34]. There is a chance that EGFR and HER2 are chosen over HER4 proteins for dimerization with HER4 proteins when EPRs are involved. Accordingly, the possibility of forming HER4 dimers is greater in cells expressing the EGFR and HER2 along with them, compared to cells expressing HER4 alone. In other words, the sensitivity of the HER4 protein for EPR increases in the presence of EGFR or HER2. Conversely, betacellulin enhances greater levels of HER4 phosphorylation compared to EPR and does not require the available different proteins to have high-affinity hormone-receptor interactions [35,36,37].

45K heparin-binding glycoprotein (p45) has similar characteristics to the heregulin proteins that work on HER4 receptor, i.e., proteins’ amino terminal sequence induces differentiation of breast cancer cells and has the ability to activate tyrosine phosphorylation in MDA-MB-453 cells [36]. Other EGFR ligands including amphiregulin, EGF and transforming growth factor-α demonstrated their low receptor stimulatory effects. Further studies are currently being performed to explore the role of the newly discovered EGFR agonist, epigen, in inducing receptor activation [31,32].

## 5. Physiological Roles and Functions of HER4

In many human tissues, both adult and fetal, a survey of HER receptor expression found that HER4 is widely expressed. HER4 plays an important role in different tissues like the heart, nervous system and endocrine system (especially the mammary glands) [2]. HER4 plays an essential role during embryonic development in addition to its involvement in cardiac development [38,39]. HER4’s role in cancer is not well understood, with studies supporting anti-proliferative role of HER4 especially in breast cancer, as well as studies supporting proliferative role can be found in the literature. Their differences can be attributed to HER4’s complicated biology and variety of ligands that can activate it, different dimerization partners, and different downstream pathways affected by HER4 [2].

## 6. HER4 Relationship to Different Cancers

### 6.1. HER4 and Colorectal Cancer (CRC)

According to World Health Organization (WHO), colorectal cancer (CRC) was the third most common type of cancer and the second most common cause of cancer-related death worldwide in 2018 with 1.80 million cases and 862,000 deaths. The peak occurrence occurred in individuals between the ages of 60–79. One of the most critical methods to indicate the early likelihood of CRC is the expansion of the tumor’s progression at the time of the diagnosis; depending on the invasion level of the tumor and the spread level to the regional lymph nodes, the prognosis can be worse [40]. Recently, studies aimed to identify possible molecular pathways involved in the progression and development of the pathogenesis of the disease to further enhance diagnostic and therapeutic modalities [41].

Several proteins have been identified and correlated with the development of CRC [42]. Amongst the widely studied proteins are HER proteins. A particular interest has been generated in the effect of HER4 expression in the progression of tumorigenesis in CRC. The effects of several therapeutic entities in the regulation of CRC through their action on these receptors have been investigated [42,43].

It has been found that HER4 proteins protect colon epithelial cells from the tumor necrosis factor (TNF) induced apoptosis by binding to epithelial cells to express anti-apoptotic and cell-protective effects when they are activated [42]. Studies suggested that HER4 overexpression in colorectal cancer is not caused by gene duplication, but due to changes occurring at the transcriptional level or changes related to protein stability [44]. Accordingly, HER4 overexpression is not the first cause of oncogenesis in colorectal cancer.

Additional analysis showed that lower HER4 promoter activity could increase the risk of colorectal cancer [44,45]. Data showed that elevated levels of messenger RNA, including the premalignant adenoma or proteins associated with HER4, are involved in CRC tumorigenesis. Henceforth, low levels of HER4 proteins expressed in a poorly differentiated colorectal cancer cell line caused impairment in its anchorage-independent growth, which is related to different malignant phenotypes [46]. Moreover, evidence suggests that HER4 overexpression may contribute with wingless related integration site (WNT) signaling to boost the human colonocytes growth [45].

HER4 proteins’ role has conflicting data on the CRC and its role in proregression and tumor growth. Currently, there are no ideal data explaining and showing its discrepancies. A couple of potential explanations for the discrepancy might be rationalized. HER4 can promote its own expression in a few systems [47]. One of the theories indicates that the role of HER4 in CRC differs among subtypes [48,49].

In summary, studies show that the HER4 receptor is over-expressed at the protein levels and mRNA in colorectal cancer. High HER4 levels are contributing to the activation of phosphatidylinositol 3-kinase (PI3K) and the EGFR pathways along with cyclooxygenase-2 (COX-2) expressions. These results indicate that HER4 can be a valid therapeutic agent and a possible target in CRC and other epithelial-based malignancies [46].

### 6.2. HER4 and Lung Cancer

According to WHO, lung cancer is the most common cause of cancer-related death worldwide in 2018 with 2.09 million cases and 1.76 million deaths. HER4 mutations in lung cancer have a low rate of clinical significance and are rare [50]. Several somatic HER4 mutations have been explained in non-small cell lung cancer (NSCLC) [51]. Characterization of nine HER4 mutations showed four different mutations, i.e., D931Y, D595V, Y285C, and K935I, along with elevated levels of ligand-induced HER4 phosphorylation levels. These mutations are localized at an important position at the HER4 kinase domain (D931Yand K935I) and in the extracellular ligand binding (Y285C and D595V). Research study has shown that they enhance the HER4 dimerization and phosphorylation whilst stimulating the proteolytic release of the HER4-ICD and enhancing the endurance of 3TE cells when serum is absent [52]. Specific HER4 polymorphisms are linked with a higher risk of lung cancer, i.e., (SNPs rs6747637, rs6740117 and rs6742399), according to that, HER4 variants may be a risk factor to lung cancer development. These data require further studies to identify their activation potential of the HER4 receptor and the possibility of having a therapeutic value of targeting mutated HER4 proteins in regard to this disease [53].

### 6.3. HER4 and Gastric Cancer

Gastric cancer is among the most widespread types of cancer globally with a significant increase in the morbidity and mortality rate during the last few decades. It is a highly heterogeneous disease. Studies and information on the involvement of HER4 in this type of cancer are still evolving. Out of 294 tested gastric cancer samples, 20 of them showed mutations in the HER4 gene [54]. One of the mutations that has occurred, HER4 p.R50C, has previously been observed in melanoma [55]. In total, 33% of the HER4 mutations in gastric cancer happened in the kinase domain, 20% in the receptor domain, indicating the influence of these mutations on kinase activity or can affect the receptor–ligand interactions [56].

### 6.4. HER4 and Hepatocellular Carcinoma

Previous reports indicate that HER4 absence in hepatocytes in mice caused an elevation in their likelihood of developing hepatocellular carcinoma (HCC), a response to a toxic stimulus like diethyl nitrosamine (DEN) [57]. Moreover, it has been reported that, compared to control hepatocytes, isolated HER4-null hepatocytes demonstrated a higher proliferation rate in vitro. HER4 activation is down-regulated in tumor samples of patients with HCC, which may be associated with a decreased cellular differentiation and poorer prognosis and quality of life [57]. This could be due to the decreased p53 activity concomitant with the inhibition of the expression of the tumor suppressor tp53inp1 upon loss of the HER4 protein. Further studies showed that loss of HER4 activity has an important role in the progression of hepatic lesions in HCC [58].

### 6.5. HER4 and Prostate Cancer 

The HER family’s role in prostate cancer progression is controversial. The HER family plays various roles in prostate cancer [59]. In prostate cancer, the expression of HER4 is upregulated [59,60,61,62]. There is a recent study stating that HER4 levels remain the same during the transition from prostate hormone-dependent to hormone-refractory cells [59]. The expression of the HER4 in hormone-sensitive tumors is correlated with a longer time of biochemical relapse. HER4 expression is high in the normal human prostate epithelium and is stated to be related to differentiation, growth arrest and tumor quelling [59,62]. Therefore, the tumor tends to be less aggressive when this receptor is expressed in tumor cells. In contrast, high HER4 expression tends to have a protective function in hormone-sensitive tumors [59].

### 6.6. HER4 and Bladder Cancer

HER family expression in bladder cancer remains uncertain [63]. However, studies showed that expression of HER4 in bladder cancer is associated with better prognosis. High expression of HER4 protects patients from the effects of high levels of other HER family members including EGFR and HER2. Patients with the highest levels of HER4 had better survival rates compared with patients with lower levels of HER4 expression [64]. Statistically important associations were shown by nuclear HER4 expression, high histologic level and advanced tumor process with non-papillary tumors. Cytoplasmic expression of HER4 was associated with good prognosis [65].

### 6.7. HER4 and Ovarian Cancer

Limited information and data are present on HER4 impact on ovarian cancer. However, high expression of HER4 was observed at high prevalence of ovarian cancer. HER4 expression was higher in tumor specimens for those with an incomplete response to chemotherapy compared to the complete response. HER4 isoforms enable or suppress downstream molecular pathways that may have similar or opposing roles in developing chemotherapeutic agent resistance. The expression of HER4 was observed mainly in the tumor cell cytoplasm, and in the ovarian cancer cell lines, it is membranous and cytoplasmic. In ovarian cancer cell lines, higher levels of expression than normal cells were reported. Compared with control tissue, HER4 expression was substantially high in the ovarian serous carcinoma specimens [66]. On the other hand, analysis of functionally different isoforms will complicate HER4 cancer biology [66,67].

### 6.8. HER4 and Breast Cancer

HER receptor family plays a role in mammary epithelial cell growth, and also in malignant transformation and tumor progression [3]. HER4 contributes to the growth and differentiation of a strictly regulated spatiotemporal expression of the mammary gland [3,68]. The significance of HER4 in breast cancer was understood in a series of experimental tests, but the results were contradictory, indicating that HER4 has both oncogenic and tumor suppressive roles [69,70]. Various studies investigating HER4’s carcinogenic function have shown that HER4 expression is usually associated with hormone receptor positivity status, including estrogen and progesterone receptors, HER2 negativity, well-differentiated phenotype, and favorable outcome [71,72,73,74]. In addition, normal HER4 gene expression and overexpression were claimed to be related to shorter relapse-free survival in comparison to patients with low HER4 gene expression [75] and unfavorable clinical outcome in patients with overexpression of HER4 [76]. Overexpression of HER4 increases the growth of human breast cancer cells by supporting a role in promoting growth [77,78] and changes mice mammary epithelial to form tumors [79].

NRG-1 increases the ratio of cells in the G2/M phase of the cell cycle in HER4-positive but not HER4-negative breast cancer cells and in comparison to HER4/HER2 or HER3/HER2 heterodimers, the G2/M expression checkpoint protein BRCA1 increases, indicating that HER4 homodimers can initiate this mechanism [80]. Activation of HER4 by NRG-1 thus delays mitosis and reduces breast cancer cell proliferation. Interestingly, HER4 mRNA expression has been found to correlate with BRCA1 mRNA expression in human breast cancer samples and requires BRCA1 activity for NRG-1-mediated breast cell growth inhibition, as shown by in vitro and in vivo BRCA1 knockdown studies [80]. These results suggest that by inducing a G2/M checkpoint via a still unknown mechanism involving BRCA1, HER4 impairs the proliferation of breast cancer cells [2]. Other researches have also shown that HER4 activity can induce cell death via mitochondrial accumulation of HER4 in breast cancer-derived cells [81] and the interaction between the BAK pro-apoptotic protein and the BH3- like HER4 domain [2].

In 1993, HER4 was initially cloned from the MDA-MB-4533 line of human breast cancer cells. T-47D, MCF-7, MDA-MB-330, MDA-MB-361, and BT-474 are few other breast cancer cell lines expressed by HER4 [13]. In breast cancer cells, HER4 expression is low compared to EGFR and HER2 expression. Multiple researches have investigated HER4 expression at the protein level in clinical breast cancer through immunohistochemistry with various anti-HER4 antibodies recognizing either the *N*- or *C*-terminus of HER4 or the reverse transcription mRNA-PCRRA-level (RT-PCR). Upregulation of HER4 expression has been identified in 7 to 29% of breast cancers, while in 18 to 75% of cases, downregulation of HER4 expression has been observed. The majority of these studies have reported up- and downregulation of HER4 simultaneously. These results suggest that HER4 can be found in breast cancer tissue in vivo in both overexpression and downregulation [82].

In the literature, relatively high HER4 expression has been correlated with estrogen receptor-positive, low-grade, and slowly proliferating breast cancers, while in oestrogen receptor-negative cases, expression of HER4 tends to be downregulated [82].

A study demonstrated that serum HER4 ectodomain concentrations can be measured from ELISA clinical samples, suggesting a different new bioassay for the evaluation of HER signalling [83].

### 6.9. HER4 and Pancreatic Cancer

HER4 expression in pancreatic cancer is not well understood [84] but it tends to be low in human pancreatic cancers [2]. Moreover, in the early stages of pancreatic cancer, HER4 transcription is diminished, implying that the lack of HER4 expression can be a requirement for tumorigenesis [85]. In pancreatic cancers, higher HER4 expression was also found to correlate with favorable staging [86].

Later studies reported that HER4 is expressed predominantly in the exocrine pancreas duct system and, to less extent, in the cancerous cells of several human pancreatic adenocarcinomas. Moreover, HER4 mRNA expression declines in non-metastatic stages of pancreatic cancer and approaches levels similar to normal control levels in advanced diseases [87].

Tissue obtained after pancreaticoduodenectomy in one study showed that in the normal pancreas, HER4 is highly expressed, but in some cancers the expression is lost [84].

### 6.10. HER4 and Brain Cancer

Glioblastoma multiforme (GBM) is the most prevalent central nervous system cancer, which is the most common, severe, and difficult to treat [88]. Studies of HER family members show that HER4 is one of the most common proteins in GBM [89]. HER4 mRNA levels were observed to be lower than in normal brain samples, and HER4 protein was found to be widely expressed in GBM, but not related with survival [90].

CCLE data suggests a role of HER4 as a tumor suppressor, by showing copy number loss of HER4 gene through several cell lines of GBM. However, the same variants have been shown to occur at about the same frequencies in the general population [88]. Though GBM has an average low level of HER4 expression, in 11 % of cases, high pHER4 expression was found to be present and associated with reduced survival than no pHER4 expression [90]. Therefore, increased HER4 activity may have prognostic and/or therapeutic effects, considering the low levels of HER4 mRNA in GBM [2].

### 6.11. HER4 and Melanoma

HER4 is one of the most mutated tyrosine kinase in melanoma. Thus, this receptor plays a crucial role in malignant melanoma. HER4’s significance in cancer is debatable due to the receptor’s dual oncogenic/tumor-suppressive properties. The findings of the studies indicate that HER4 is oncogenic in malignant melanoma. This is corroborated by the fact that 19% of patients with metastatic melanomas carry a HER4 mutation. As a result of this, HER4 has been proposed as a potential therapeutic target in melanoma [91,92,93,94].

### 6.12. HER4 and Endometrial Cancer

Endometrial cancer is the most prevalent female genital tract cancer, affecting mostly postmenopausal women. Endometrial cancer hits around 2% to 3% of all women throughout their lifetime [95]. In a study examining the expression of the epidermal growth factor system in endometrial cancer, HER4 was found to be overexpressed in endometrial cancer higher than in healthy postmenopausal endometrium. The expression of the HER4 receptor showed no association with tumor grade and stage, nor with the disease’s outcome [96].

### 6.13. HER4 and Osteosarcoma

Osteosarcoma is the most prevalent bone tumor in adolescence. It is characterized by fast progression, metastasis, and poor prognosis [97,98]. In a study investigating the pathway in which HER4 promotes osteosarcoma, HER4 proteins were found to be highly expressed in osteosarcoma cells. It was found that increased HER4 levels highly promoted proliferation, metastasis, and tumor progression in vitro and in vivo. High levels of HER4 were associated with increased expression of other protein kinases that are responsible for osteosarcoma progression, thus, HER4 is considered a potential target for new therapeutic modalities for the treatment of osteosarcoma [99].

## 7. HER4 Inhibitors

Kinase inhibitors are categorized based on kinase conformation when inhibitors bind (e.g., type1, type 1_1/2_, and type 2), and type of inhibition (e.g., reversible or irreversible). Most of the reported drugs that inhibit HER4 are mainly covalent inhibitors, as they bind to the ATP-binding site, covalently/irreversibly making them stronger than reversible drugs. However, the risk of more serious side effects is higher [5].

### 7.1. Quinazoline Inhibitors

In these derivatives, the quinazoline ring nitrogen atom(s) together with the attached NH are important to mimic the interaction of ATP molecule with the kinases hinge region. Michael acceptor-possessing molecules are designed to act as irreversible inhibitors.

#### 7.1.1. Allitinib (AST-1306) 



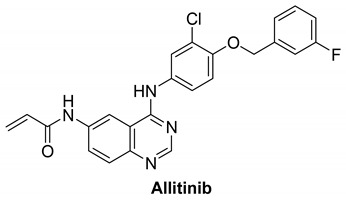



Allitinib is a novel anilino-quinazoline compound that is orally active and has been synthesized based on the lapatinib chemical structure [100]. It is an irreversible inhibitor of EGFR, HER2 and HER4 with IC_50_ of 0.5, 3, and 0.8 nM, respectively [101]. It is 5 to 15 times more potent compared to dacomitinib and afatinib. In human tumor xenograft models expressing or overexpressing HER family members, allitinib showed antitumor activity, especially in those with HER2 overexpression or EGFR T790M mutant tumors [101]. It binds irreversibly to Cys797 and Cys805 in the catalytic domains of EGFR and HER2, respectively [102]. It has two hydrogen bond donors, six hydrogen bond acceptors, and seven rotatable bonds [103].

It was investigated that allitinib significantly inhibited the proliferation of gastric cancer cells. In vivo, it controls the HER4-PI3K/Akt signaling pathways [4,104].

#### 7.1.2. Poziotinib (HM781-36B) 



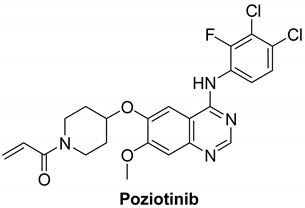



Poziotinib is an oral, third-generation, quinazoline-based inhibitor of epidermal growth factor receptors (EGFR, HER2, and HER4) that Hanmi Pharmaceutical has developed, currently being investigated in phase II clinical trials for breast and non-small cell lung cancer treatment [105]. It irreversibly inhibits HERs in vitro with IC_50_ values of 3.2, 5.3, 23.5 nM for EGFR, HER2 and HER4, respectively [106]. At the C6 position, it has a functional α,β-unsaturated carbonyl group like other irreversible EGFR inhibitors that makes covalent modifications to the active site of the EGFR kinase domain [107].

Poziotinib was terminated in 3 clinical studies for NSCLC (patients having EGFR or HER2 exon 20 insertion mutation), breast cancer (In HER2+ breast cancer patients, poziotinib in combination with T-DM1) and adenocarcinoma of lung stage IIIB and IV [108,109,110]. It is currently active but not recruiting in three clinical studies for solid tumor, breast cancer (HER2+ metastatic BC) and metastatic breast cancer (HER2+ patients with recurrent stage IV cancer who have had at least two previous HER2-directed treatments) [111,112,113]. Moreover, it is recruiting in five clinical studies and completed seven studies in conditions including metastatic breast cancer, HER2 gene mutation, adenocarcinoma lung stage IV, advanced solid malignancies, HER2+ advanced gastric cancer, increased drug resistance and advanced solid tumor. Unfortunately, there are three studies with unknown status [114].

HER4 is highly expressed in ovarian cancer stem cells. Poziotinib inhibited ovarian CSCs from sphere formation, viability, and proliferation. In addition, it triggered growth 1 (G1) cell cycle arrest and apoptosis. Furthermore, poziotinib decreased the stemming of CSCs and interfered with the pathways of Wnt/β-catenin, Notch, and Hedgehog that lead to CSC self-renewal [115]. Ovarian cancer stem cells have been scientifically shown to survive conventional chemotherapy [116]. Although ovarian CSCs have not been clearly understood in this respect, a small population of chemo-resistant cancer cells may have the properties of cancer stem cells and play an important role in recurrence [117,118]. To interrupt cancer stem cell properties and stem cell signaling pathways, a novel approach is required [115].

**Figure 3:**
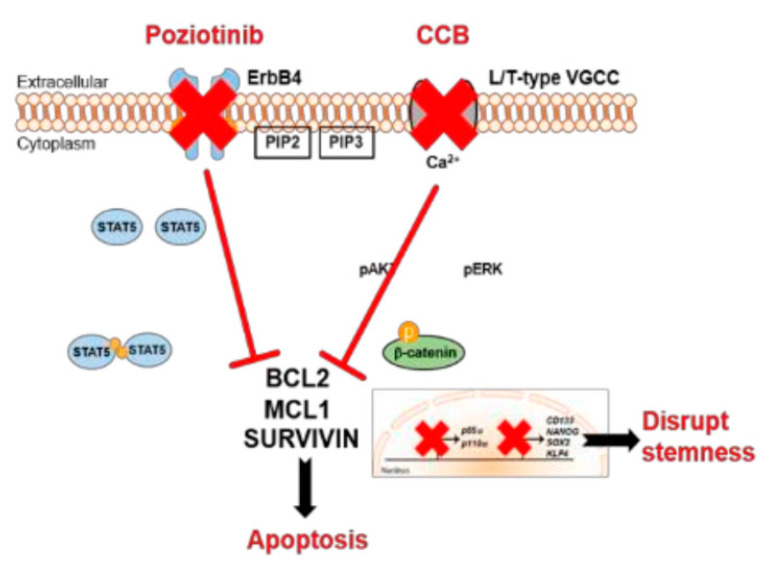
Mechanisms of action of poziotinib and manidipine in ovarian CSCs [115].

It was shown using Chou–Talalay method that combination therapy of poziotinib with manidipine (a dihydropyridine calcium antagonist) showed a synergistic effect in inhibiting ovarian CSCs more than in ovarian cancer cells as shown in Figure 3 [115]. Combination therapy with poziotinib and manidipine inhibited the expression of stem cell markers, especially CD133, NANOG, and KLF4. The two drugs also inhibited the phosphorylation of STAT5, AKT, and ERK, which are involved in CSC self-renewal and β-catenin nuclear translocation [115].

Poziotinib and manidipine combination therapy showed synergistic effect in inhibiting ovarian CSC by inhibiting HER4 and calcium channel mediated STAT5, AKT and ERK signaling [115].

#### 7.1.3. Dacomitinib (PF-00299804)



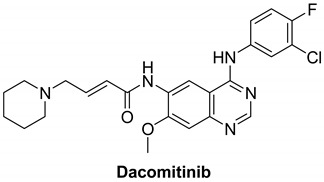



Dacomitinib is a second-generation RTK inhibitor used for the treatment of metastatic NSCLC (development phase three), gastric cancer (development phase one/two), head and neck cancer (development phase one/two) and glioblastoma (development phase one/two) [119]. Because of acquired tolerance to first-generation EGFR tyrosine kinase receptor inhibitors, the clinical development of second-generation EGFR tyrosine kinase inhibitors assisted in bypassing many pathways of resistance to first-generation EGFR tyrosine kinase receptor inhibitors. The EGFR kinase domain forms permanent covalent bonds with most second-generation tyrosine kinase receptor inhibitors. Dacomitinib has the ability to inhibit EGFR, HER2, HER3, and HER4 in an irreversible manner [120].

Dacomitinib possesses aminoquinazoline as its adenine pocket moiety. It has a 3-chloro-4-fluoro substituted phenyl that enters a hydrophobic pocket and a Michael acceptor that forms a covalent bond with cysteine [120]. Both HER homodimers and heterodimers are prevented from signaling to the cell. Dacomitinib reduces EGFR signaling in tumors/cells with multiple EGFR mutations but has only a minor effect on tumors with Kirsten rat sarcoma viral mutations, according to preclinical studies. Dacomitinib inhibits the ErbB family of kinases irreversibly and selectively, with IC_50_ values of 6, 45.7, and 73.7 nM against EGFR, HER2, and HER4, respectively.

Second-generation irreversible kinase inhibitors such as afatinib and dacomitinib were initially discovered to be a candidate against diagnosed epidermal growth factor receptor mutated lung cancer. They struggled to resolve T790M-mediated resistance in patients, as they have in monotherapy. The concentrations in which these irreversible inhibitors bypass T790M activation are not preclinically feasible in humans due to the dose-limiting toxicity associated with nonselective inhibition of wild-type EGFR. T790M resistance is induced by these inhibitors, meaning that they are less effective against T790M. As a result, there is a significant unmet need for an inhibitor that can more effectively target T790M tumors while leaving wild-type EGFR alone. As a result, “third-generation” inhibitors have been developed [121,122].

#### 7.1.4. Lapatinib



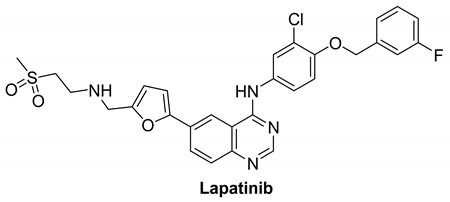



Lapatinib is an EGFR double tyrosine kinase inhibitor that is taken orally [123]. It is a type I inhibitor that possesses 4-anilinoquinazoline scaffold. By competing for HER2 and EGFR’s ATP binding sites, lapatinib reversibly inhibits their activation. Lapatinib is used to treat advanced or metastatic breast cancer, as well as patients with brain metastases [123]. It interacts with HER4 in a similar way to the EGFR kinase [1]. Lapatinib inhibits EGFR, HER2 and HER4 with IC_50_ values of 10.8, 9.2, and 367 nM, respectively [124].

Lapatinib was terminated in one clinical study as protocol would not be able to approach stated accrual. Tumor cells from around 20% of melanoma patients have a particular mutation of a gene involved in producing HER4, and variations in this gene have been related to cancer. Lapatinib has been shown to substantially delay the growth of melanoma cells with this HER4 gene mutation. Further investigation on whether lapatinib can be beneficial for the treatment of melanoma is needed [5].

For patients with metastatic, EGFR antibody-resistant bowel cancers, a combination of trastuzumab and Lapatinib looks promising, but it is unclear whether the same combination is better than an EGFR antibody for patients who have never been treated for metastatic bowel cancer yet [5].

**Figure 4:**
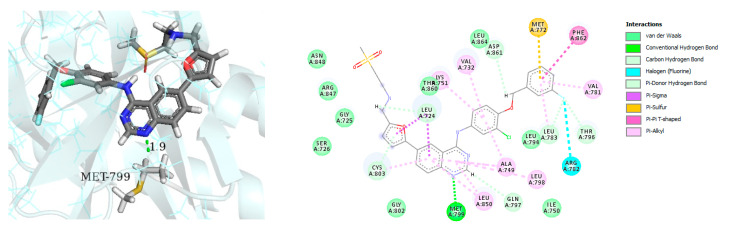
Best-docked pose of Lapatinib within the HER4 (ErbB4) active site, where hydrogen bond interactions are shown as green dashed lines (**left panel**) and its corresponding detailed 2D-interactions map (**right panel**). This docking study was conducted by us, no copyright issues.

Binding mode analysis of Lapatinib within the HER4 active site revealed a number of important interactions: the compound was able to form strong hydrogen bond interaction (distance, 1.9 Å) with the corresponding amino acid residue Met-799 (Figure 4 left panel). Other important interactions include the formation of pi–pi stacking between the 3-fluorobenzyloxy terminal arm and the residue Phe-862, formation of pi–sigma interactions between the furan and quinazoline rings and the corresponding residue Leu-724 (Figure 4 right panel). 

#### 7.1.5. Afatinib (BIBW2992)



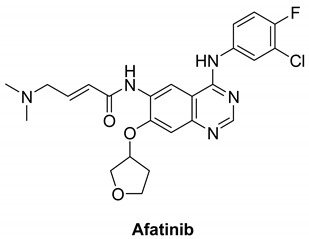



EGFR, HER2, and HER4 have an effect on tumor cell proliferation and are presumed to be overly expressed in many cancer cells [125]. Five prospective clinical trials had evaluated afatinib’s effectiveness in patients with advanced metastatic non-small cell lung cancer. Afatinib is an anilino-quinazoline derivative and works by inhibiting the epidermal growth factor receptor family by utilizing antineoplastic activity. It is used to treat NSCLC. It is an irreversible inhibitor that binds by covalent bonds to the intracellular tyrosine kinase domain of the epidermal growth factor receptors, i.e., EGFR, HER2, HER4, and a few epidermal growth factor receptor mutants such as the ones caused by exon 19 and 21 (L858R) deletion and substitution mutations, respectively [126].

In vitro, Afatinib inhibits HERs irreversibly with IC_50_ values of 0.4, 10, 14, and 1 nM against EGFR (wt), EGFR (L858R), HER2, and HER4, respectively. This is presumed to lead to inhibiting tumor growth and cause angiogenesis in the tumor cells that overexpress RTKs. In addition, trials showed that afatinib improved the overall survival rate and showed inhibition against T790M-mutant EGFR gatekeeper, which is well-known to be resistant to first-generation EGFR inhibitors [127]. Therefore, afatinib along with erlotinib and gefitinib, has been approved as a first-line treatment for patients with metastatic NSCLC [128,129].

Patients are advised to continue the therapy until they experience symptomatic disease progression. However, disease progression does not prevent the physicians from using another agent in its class or possible future reuse of the treatment. Afatinib is known to be correlated with an elevation in serum aminotransferase levels throughout the therapy causing acute liver injury and in rare cases mortality [130].

#### 7.1.6. Canertinib (CI-1033) 



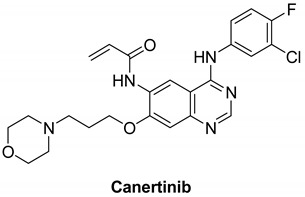



Canertinib is an oral pan-HER inhibitor that inhibits all four HER receptors [131]. Canertinib is composed of an aminoquinazoline ring in the adenine pocket, a morpholino ring in the solvent field, and an acrylamide moiety as a Michael acceptor to form a covalent bond with the cysteine residue. 

Canertinib irreversibly inhibits the enzymatic activities of EGFR, HER2, and HER4 with IC_50_ values of 0.8, 19, and 7 nM, respectively [132]. Canertinib also has poor clinical results, and the occurrence of side effects including diarrhea and rash in patients with advanced NSCLC has reduced its clinical use [132]. Canertinib has three completed clinical studies including treating patients with metastatic (stage IV) breast cancer (phase II), evaluating the adequate protection and effective dose of canertinib in combination with paclitaxel and carboplatin in patients with advanced NSCLC (phase I) and an open-label review of canertinib as a single agent in patients with severe non-small cell lung cancer [133,134,135]. Pfizer agreed to stop developing the drug in 2015 because of concerns about its safety and risk/benefit ratio. In the United States, there are currently no canertinib breast cancer studies ongoing [131].

### 7.2. Quinoline Inhibitors

#### 7.2.1. Neratinib (HKI-272)



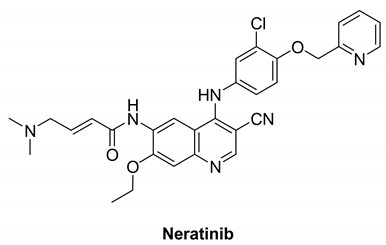



Neratinib is made up of an aminoquinoline ring that binds in the adenine pocket, a pyridine substitution that occupies the solvent regions, and a Michael acceptor, which is needed for covalent inhibitors [136].

Neratinib is an irreversible EGFR inhibitor that has been developed and tested in clinical studies. Neratinib treats some forms of cancer, it inhibits the oncogenic intracellular signaling pathways of EGFR, HER2, and HER4, inhibiting autophosphorylation and activation [137]. Neratinib inhibits the kinase activity of EGFR, HER2, and HER4 (IC_50_ values of 92, 59, and 19 nM, respectively) [138].

Neratinib is currently recruited in one clinical study. This phase I trial focuses on the adverse effects and preferred dose of Neratinib in combination with everolimus, palbociclib, or trametinib in patients who have solid tumors with EGFR mutations/amplification, HER2 mutations/amplification, HER3 and HER4 mutations, or KRAS mutations that affect other body parts and are refractory to treatment (advanced or metastatic). Neratinib, palbociclib, and trametinib can inhibit tumor cell growth by inhibiting certain enzymes required for cell growth. Chemotherapy drugs like everolimus function in a number of ways to stop tumor cells from growing, including destroying them, preventing them from dividing, and preventing them from spreading. In the treatment of solid tumors, Neratinib combined with everolimus, palbociclib, or trametinib may be more effective than neratinib alone [138].

#### 7.2.2. Pyrotinib (SHR1258) 



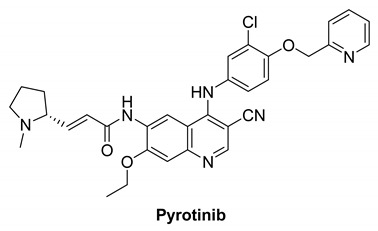



Pyrotinib is a second-generation pan-HER receptor tyrosine kinase inhibitor that targets EGFR, HER2, and HER4. It is an irreversible inhibitor and well absorbed [139]. Its IC_50_ values are 5.6 and 8.1 nM against EGFR and HER2, respectively [140,141]. Clinical trials of pyrotinib/vinorelbine combination therapy of brain metastases from HER2-positive metastatic breast cancer are recruited [142].

Pyrotinib is being examined in another clinical study that is divided into two sections. Investigators will assess the protection and tolerability of pyrotinib plus capecitabine combined with brain radiotherapy in a phase Ib trial. Investigators will study the data after the phase Ib part is completed and determine whether to include this patient before starting the phase II part. Investigators will determine the effectiveness of pyrotinib and capecitabine combined with brain radiotherapy in patients with HER2 positive breast cancer with brain metastases in the phase II portion of the research [139].

Pyrotinib and inetetamab demonstrated high standard anti-tumor efficacy and acceptable protection and optimized ADCC, respectively, in a recruiting study. In order to assess the effectiveness and safety of inetetamab in combination with pyrotinib and chemotherapy for treatment of HER-positive metastatic breast cancer, a phase II single-arm clinical trial is intended to be carried out [143].

### 7.3. Other Inhibitors

#### 7.3.1. Ibrutinib (PCI-32765)



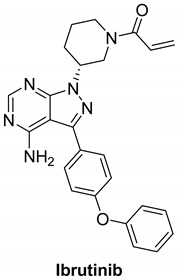



Ibrutinib is an orally delivered, irreversible first-generation Bruton’s tyrosine kinase inhibitor (BTK). BTK belongs to the cytoplasmic non-receptor tyrosine kinase class of tyrosine protein kinases (TEC) and is found primarily in hematopoietic cells. Mantle cell lymphoma (MCL), chronic lymphocytic leukemia (CLL), Waldenstrom macroglobulinemia (WM), Marginal zone lymphoma (MZL) and chronic Graft-versus-host disease (GVHD) which are all indications for ibrutinib [144,145].

BTK is a key component of the B-cell receptor signaling pathway and a mediator of pro-inflammatory signals [146]. BTK inhibition can be a treatment tool for B-cell malignancies and autoimmune conditions. BTK is one of 11 tyrosine kinases, including TEC family kinases, EGFR, HER2, HER4, BLK, and JAK3, which all share a conserved cysteine residue adjacent to the ATP-binding site, allowing covalent inhibition by tyrosine kinase inhibitors. Ibrutinib binds covalently to the cysteine-481 at the active site of BTK, inhibiting kinase activity for more than 24 h with an IC_50_ of 0.5 nM [147]. Ibrutinib inhibits HER phosphorylation and the differentiation of HER2 breast cancer cells, implying that it may be used to treat breast cancer [148].

A recent study demonstrated that using the Nucleic Acid Programmable Protein Array (NAPPA) functional protein microarray platform, researchers discovered that HER4 is a promising candidate for ibrutinib. It was discovered that HER4-expressing cells react to ibrutinib via the WNT pathway and that inhibits some WNT activating ligands which help boost ibrutinib reaction. Findings indicate that ibrutinib could be used to treat HER4-expressing cancers in addition to B-cell malignancies, either alone or in conjunction with WNT inhibitors [149,150,151].

#### 7.3.2. AC-480 (BMS-599626)



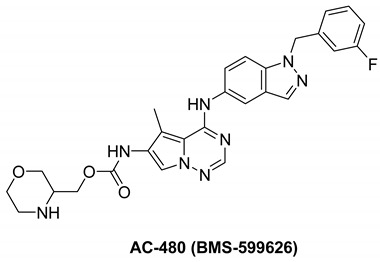



AC-480 is a small molecule and a pyrrolotriazine analogue that is orally active and has been synthesized by Bristol Myers Squibb Company [152,153]. It reversibly inhibits tyrosine kinase receptors EGFR, HER2, and HER4 with IC_50_ of 22, 32, and 190 nM, respectively, and, to a lesser extent, HER3 [152,154]. AC480 is an ATP competitive inhibitor in EGFR inhibition, according to kinetic studies. AC480, on the other hand, inhibited HER2 via an ATP non-competitive mechanism. Therefore, it acts as a mixed-type inhibitor with Ki values equal 2 and 5 nM, respectively [152].

AC-480 inhibits heterodimerization of EGFR and HER2 receptors, this adds an alternative tumor-inhibiting mechanism in which receptor co-expression and heterodimerization contribute to tumor development. The preclinical studies encourage the development of AC-480 for cancer treatment in humans [152]. AC-480 had completed four clinical studies which are phase I study in patients with HER2-expressing advanced solid malignancies, phase I study in treating patients with metastatic solid tumor, phase I study in patients with advanced solid malignancies, including malignancies that express HER2 at the maximum tolerated dose and/or recommended, phase I dose and pharmacokinetic study of AC480 in patients with recurrent malignant glioma have all been completed [155,156,157,158] and safety study for intravenous (IV) AC-480 to treat advanced solid tumors has been withdrawn [159,160].

When comparing the effects of BMS-536924 in addition with AC-480 to the effects of the single agents alone, repeated experiments indicated that the antiproliferative effect of the combination was synergistic [160].

In the five ovarian cancer cell lines that displayed synergistic antiproliferative activity with BMS-536924 and AC480 (BMS-599626), there was a hypothesis that receptor expression and/or phosphorylation modulation occurred. All the five cell lines showed signs of increased HER receptor signaling activity after treatment with BMS-536924. AC480 inhibited the increase in HER receptor signaling in all ovarian cell lines. By Western blotting, there was no observable activation of HER4 in any of the ovarian cell lines [160].

**Figure 5:**
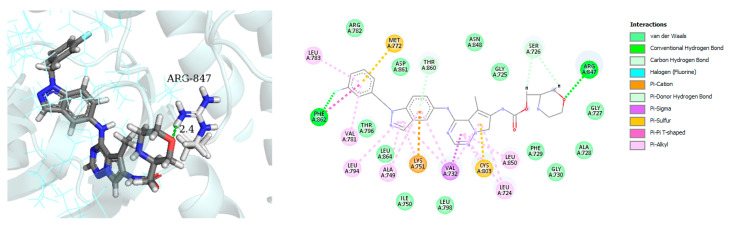
Best-docked pose of compound AC-480 within the HER4 (ErbB4) active site, where hydrogen bond interactions are shown as green dashed lines (**left panel**) and its corresponding detailed 2D-interactions map (**right panel**). This docking study was conducted by us, no copyright issues.

The best docked conformation of compound AC-480 (BMS-599626) within the HER4 (ErbB4) active site revealed strong hydrogen bond (distance, 2.4 Å) formed between the morpholino terminal moiety and the corresponding Arg-847 amino acid residue (Figure 5 left panel). Furthermore, the compound was able to secure large network of weak interactions including van der Waals, pi–sulfur, pi–cation and pi–alkyl interactions as illustrated in (Figure 5 right panel)

#### 7.3.3. Compounds I and II



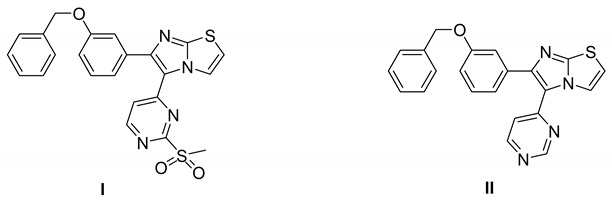



These two compounds are first-in-class imidazo[2,1-*b*]thiazole-possessing potent and selective inhibitors of HER4 kinase. This recent study reported selective HER4 inhibitors for the first time, unlike the other previously mentioned inhibitors that are non-selective kinase inhibitors. The IC_50_ values of compounds **I** and **II** against HER4 kinase in cell-free kinase assay are 15.24 and 17.70 nM, respectively. Both exerted relative selectivity against HER4 upon testing against a panel of 63 kinases. Both compounds were tested for antiproliferative activity against NCI-60 cancer cell line panel but compound **I** exerted stronger activity. The mean inhibition percentage values of compounds **I** and **II** against the 60 cell lines upon testing at 10 µM concentration are 36.62% and 21.58%, respectively. Compound **I** was selected for five-dose testing to measure its IC_50_ values against the NCI-60 cell lines, but compound **II** was not selected. Compound **I** exerted high potency against several cell lines of different cancer types. The most sensitive human cancer cell lines to compound **I** are SK-MEL-5 (melanoma), MOLT-4 (leukemia), MDA-MB-468 (breast), UO-31 (renal), DU-145 (prostate), and HCC-2998 (colon) with IC_50_ values of 0.51, 1.02, 1.04, 1.55, 1.67, and 1.78 µM, respectively. It is more potent than sorafenib and ibrutinib, reference standard kinase-inhibiting anticancer agents, against most of these cell lines. Compound **I** was further tested in whole-cell kinase assay against T-47D breast cancer cell line rich in HER4 kinase. The compound showed ability to cross the cell membrane and inhibit HER4 inside the cells. Its IC_50_ value in this assay is 3.30 µM, which is less than its antiproliferative activity against the same cell line (4.08 µM). Moreover, compound **I** exerted other merits such as weak inhibition of hERG, CYP2D6, and CYP3A4 in addition to weak potency against WI-38 normal cells.

Structure–activity relationships of these two compounds and their derivatives against HER4 kinase and the NCI-60 human cancer cell line panel are similar. Pyrimidinyl ring carrying mesyl group is the best option for activity. Pyrimidine ring lacking methylsulfonyl group (e.g., compound **II**) is still favorable for HER4 kinase inhibition. Replacement of this moiety with unsubstituted phenyl or mesyl-substituted phenyl led to a loss of activity. In addition, imidazo[2,1-*b*]thiazole nucleus is more optimal for activity than the isosteric imidazooxazole. Regarding the benzyloxy substituent, its presence in *meta* position is more favorable for activity than *para* position. Extension of benzyl (e.g., phenethyl, 4-fluorophenethyl, or phenylethanone) led to less activity. Furthermore, *p*-fluoro-substituted benzyl analogue of compound **I** is 47-fold less potent against HER4 in cell-free assay (IC_50_ = 719 nM). Replacement of benzyl substituent with hydrophilic moiety led to complete loss of activity against HER4 kinase and cancer cell lines.

**Figure 6:**
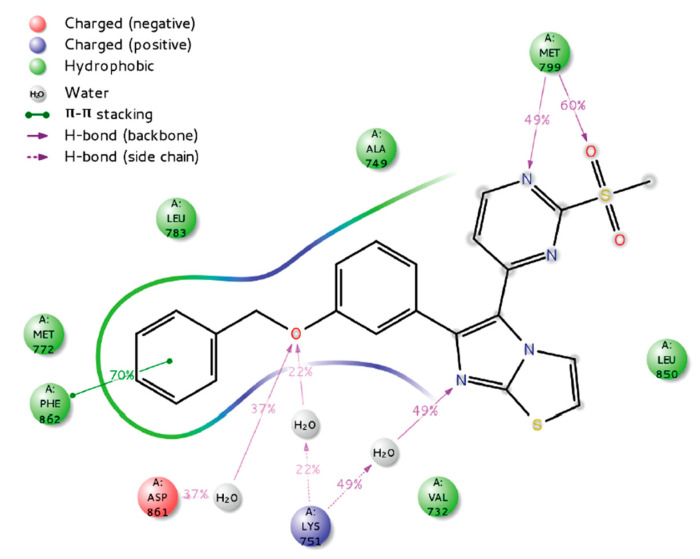
Putative binding interactions of compound **I** with HER4 active site [161]. Reprinted with permission from ref. [161]. Copyright 2021 Elsevier.

Docking and molecular dynamic simulation of compound **I** bound to HER4 active site revealed the formation of five hydrogen bonds and one hydrophobic interaction (Figure 6). Pyrimidinyl nitrogen, sulfonyl oxygen, imidazo[2,1-*b*]thiazole nitrogen, and benzyloxy oxygen accept the five hydrogen bonds. In addition, the benzyl ring forms hydrophobic interaction with Phe862 [161].

Table 1 summarizes the structures, potency, biological activities, and clinical trials (in case of clinical candidate) of the reported HER4 inhibitors.

## 8. Conclusions

With the ever-growing need for new treatments for the management of cancer, new therapeutic targets have increasingly become an absolute necessity for enhancing the patient’s quality of life. HER4 is a tyrosine kinase whose contribution to cancer is still controversial. Several reports highlighted the impact of its overexpression and mutation of several cancer types as reported in this article. However, other articles reported the opposite. Most of the previously reported HER4 inhibitors are non-selective or pan-HER inhibitors. The recent discovery of selective HER4 inhibitors such as the first-in-class imidazo[2,1-*b*]thiazole-based compounds **I** and **II** can be a great addition to this field. Those selective molecules can help molecular biologists and pathologists to better understand whether HER4 is a validated, potential target for cancer therapy or not. The future directions in this domain should involve lead optimization and development of more potent and selective HER4 inhibitors as well as extensive molecular biology research work to conclude whether HER4 inhibition is a potential drug target for cancer treatment or not.

## Figures and Tables

**Table 1:** Structures, potency, biological activities, and clinical trials (if any) of the reported HER4 inhibitors.

Name	Structure	Type of Inhibitor	IC_50_ against HER4 Kinase	Other Biological Activity	Status of Clinical Trials	Company
Allitinib	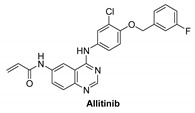	Irreversible	0.8 nM	**In vitro:** In HIH3T3-EGFR T790M/L858R cells, allitinib effectively suppresses EGFR phosphorylation. In NCI-H1975 cells with the EGFR T790M/L858R mutation, allitinib inhibits growth in a concentration-dependent manner. In vivo: In SK-OV-3 and Calu-3 xenograft models, allitinib significantly reduced tumor formation. Blocks phosphorylation of EGFR and downstream pathways. Tumors in SK-OV-3 models nearly vanish after 1 week of treatment with allitinib. Slightly inhibits the growth of tumor in HO-8910 and A549 xenograft models.	Active but not recruiting: One clinical study to analyze the efficacy and safety of anlotinib in combination with allitinib in the treatment of lung cancer.	Investigational
Poziotinib	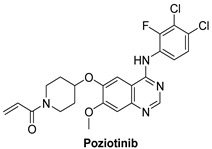	Irreversible	23.5 nM	**In vitro:** Inhibits cell growth in HER2-amplification gastric cancer cells as well as phosphorylation of EGFR and key downstream signaling cascade components including STAT3, AKT, and ERK Causes apoptosis and growth 1 cell cycle arrest in HER2 amplified gastric cancer cells by activating the mitochondrial pathway. In both HER2 amplified and HER2 non-amplified gastric cancer cells, it has synergistic effects with chemotherapeutic agents. **In vivo:** Poziotinib (0.5 mg/kg p.o.) inhibits tumor growth in nude mice carrying N87 human gastric cancer xenografts, and coadministration of Poziotinib and 5-FU induces more successful tumor inhibition. Poziotinib has shown to have excellent antitumor activity in a number of EGFR- and HER-2-dependent tumor xenograft models.	**Active but not recruiting:** Solid tumor Breast cancer Metastatic breast cancer. **Recruiting** Study to allow continued dosing and/or follow-up of patients who have had previous exposure to poziotinib, EGFR exon 20 mutant advanced nsclc Study of poziotinib in Japanese patients with NSCLC. A study of poziotinib in patients with egfr or her2 activating mutations in advanced malignancies Phase 2 study of poziotinib in patients with nsclc having egfr or her2 exon 20 insertion mutation **Terminated** 3 clinical trials. **Completed** 7 clinical trials. **Unknown status** 3 studies	Hanmi Pharmaceutical, South Korea
Dacomitinib	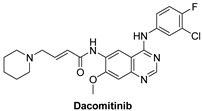	Irreversible	73.7 nM	**In vitro:** At clinically significant concentrations, dacomitinib inhibited the action of DDR1, EPHA6, LCK, DDR2, and MNK1 in vitro. In mice carrying subcutaneously implanted human tumor xenografts driven by HER family targets, including mutated EGFR, dacomitinib inhibited EGFR and HER-2 autophosphorylation and tumor development. **In vivo:** Dacomitinib showed impressive antitumor efficacy in vivo as a single agent.	**Completed:** PF-00299804 Monotherapy in Patients With HER-2 positive advanced gastric cancer. **Not yet recruiting:** An Open Label, Multicentre, Phase II Study of Dacomitinib for EGFR Mutated Non-Small Cell Lung Cancer (NSCLC) With Brain Metastases **Active, not recruiting:** Study of dacomitinib and osimertinib for patients with advanced EGFR mutant lung cancer. **Recruiting:** Dacomitinib for treatment of patients in India with metastatic NSCLC with EGFR activating mutations. **Recruiting:** Phase II study of dacomitinib in NSCLC. Dacomitinib in lung cancer with uncommon EGFR mutations A pilot study of dacomitinib with or without osimertinib for patients with metastatic EGFR mutant lung cancers with disease progression on osimertinib. Dacomitinib treatment followed by 3rd generation EGFR-TKI in patients with EGFR mutation positive advanced NSCLC. **Not yet recruiting:** Real world utilization and outcomes with dacomitinib first line treatment for EGFR mutation-positive advanced non small cell lung cancer among asian patients-a multi center chart review. **Unknown:** ARCHER1050: A study of dacomitinib vs. gefitinib in 1st-line treatment of advanced NSCLC. **Completed:** Safety and efficacy of PF-299804 (Dacomitinib), in patients with recurrent glioblastoma with EGFR amplification or presence of EGFRvIII mutation. a phase II clinical trial.	Pfizer
Lapatinib	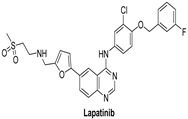	Reversible	3.67 nM	**In vitro:** Lapatinib weakly inhibits the activity of ErbB4. Lapatinib inhibits the autophosphorylation of EGFR and ErbB2 receptors in a dose-dependent manner. **In vivo:** The growth of BT474 and HN5 xenografts is significantly inhibited by oral administration of Lapatinib (100 mg/kg) twice daily in a dose-dependent manner.	**Terminated** Study as protocol would not be able to approach stated accrual.	GlaxoSmithKline
Afatinib	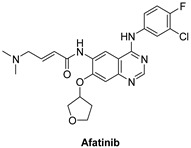	Irreversible selective inhibitor	1 nM	**In vitro:** In cell lines expressing wild-type EGFR as well as chosen EGFR exon 19 deletion mutations, exon 21 L858R mutations, or a less frequent non-resistant mutations, afatinib inhibits autophosphorylation and in some cases along with proliferation. **In vivo:** Afatinib maintains its inhibitory effects on signal transduction in vivo cancer cell development in tumors prone to reversible EGFR inhibitors, such as those with T790M mutations.	**Completed:** Afatinib Treatment for Patients with EGFR Mutation Positive NSCLC who are age 70 or older. **Terminated:** Afatinib in EGFR+NSCLC (Recurrent or Stage IV)-Patients with Poor performance Status (ECOG 2 or 3). **Completed:** Afatinib Monotherapy in Patients With ERBB-deregulated Metastatic Urothelial Tract Carcinoma After Failure of Platinum Based Chemotherapy.	Boehringer Ingelheim
Canertinib	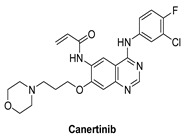	Irreversible	7 nM	**In vitro:** Canertinib alone suppresses constitutively activated Akt and MAP kinase. **In vivo:** At 5 mg/kg body weight, canertinib displays remarkable activity against A431 xenografts in nude mice.	**Unknown** Canertinib has had poor clinical outcomes, and the presence of side effects such as diarrhea and rash in advanced NSCLC patients has restricted its clinical applications.	Pfizer, development discontinued
Neratinib	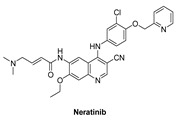	Irreversible	19 nM	**In vitro:** Neratinib weakly inhibits tyrosine kinases and Src. Neratinib displays no activity against other serine-threonine kinases. **In vivo:** Oral administration of Neratinib inhibits the development of 3T3/neu xenografts significantly. Neratinib inhibits the growth of BT474 xenografts Neratinib is also effective against SK-OV-3 xenografts	**Recruited** Phase I trial that focuses on the side effects and best dose of Neratinib in combination with everolimus, palbociclib, or trametinib in patients who have solid tumors with EGFR mutations/amplification, HER2 mutations/amplification, HER3/4 mutations, or KRAS mutations that have spread to other parts of the body and are refractory to treatment (advanced or metastatic). Neratinib, palbociclib, and trametinib can inhibit tumor cell growth by inhibiting certain enzymes required for cell growth.	Wyeth & Pfizer
Pyrotinib	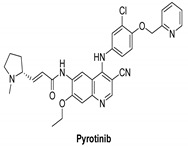	Irreversible	unknown	**In vitro:** Pyrotinib, a dual tyrosine kinase inhibitor for EGFR and HER2, has excellent in vitro potency, selectivity, and PK profiles. **In vivo:** Pyrotinib has shown to have potent anti-tumor effects in HER2-overexpressing xenograft models, as well as adequate safety windows in animals and beneficial pharmacokinetic properties in humans.	**Recruited:** Clinical study of Pyrotinib plus Vinorelbine as the therapy of brain metastases from HER2-positive metastatic breast cancer. **Running:** Pyrotinib, on the other hand, is being examined in another study, which is divided into two sections. Investigators will assess the protection and tolerability of pyrotinib Plus Capecitabine combined with brain radiotherapy in phase Ib trial.	Shanghai Hengrui Pharmaceutical
Ibrutinib	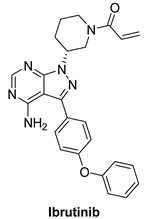	Irreversible	Unknown	**In vitro:** Ibrutinib blocked HER-amplification cell lines as well as main signalling pathways. **In vivo:** Tumor volumes in ibrutinib-responsive mouse xenograft tumors were reduced with ibrutinib therapy.	**Active, not recruiting:** A phase I/II study of ibrutinib in previously treated EGFR mutant NSCLC.	Janssen
AC-480	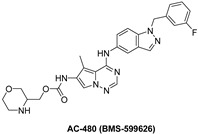	Reversible	190 nM	**In vitro:** For HER1, it works as an ATP-competitive inhibitor, and for HER2, it works as an ATP-noncompetitive inhibitor. By promoting cycle redistribution and inhibiting DNA repair, AC-480 greatly improves the radiosensitivity of HN-5 cells expressing both EGFR and HER2. **In vivo:** Inhibits Sal2 tumor growth in a dose-dependent manner. At its maximum tolerated dose of 180 mg/kg, AC-480 has potent antitumor activity in a human breast tumor KPL-4 xenograft.	**Completed:** Four clinical studies in PK study for recurrent glioma, metastatic solid tumors, advanced solid malignancies and MAD refractory. **Withdrawn:** One clinical study in safety study to treat advanced solid tumors.	Bristol Myers Squibb
Compound I	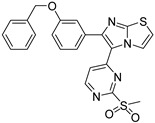	Reversible	15.24 nM	Selective HER4 inhibitor. Potent, broad-spectrum antiproliferative activity against different cell lines of several cancer types. Whole-cell HER4 kinase inhibition effect in T-47D cells. Weak inhibitor of hERG, CYP2D6, and CYP3A4.	-	Investigational
Compound II	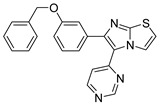	Reversible	17.70 nM	Selective HER4 inhibitor. Less active as antiproliferative agent than compound I.	-	Investigational

## Data Availability

Not applicable.

## Sample Availability

Not available from authors.

## Abbreviations

BTC	betacellulin
BTK	Bruton’s tyrosine kinase
CLL	chronic lymphocytic leukemia
COX-2	cyclooxygenase-2
CRC	colorectal cancer
DEN	diethyl nitrosamine
EGF	epidermal growth factor
EGFR	epidermal growth factor receptor
EPR	epiregulin
GBM	glioblastoma multiforme
GVHD	Graft-versus-host disease
HCC	hepatocellular carcinoma
HER	human epidermal growth factor receptor
MAPK	mitogen-activated protein kinase
MCL	mantle cell lymphoma
MZL	marginal zone lymphoma
NAPPA	nucleic acid programmable protein assays
NEU	neurogulin
NSCLC	non-small cell lung cancer
PI3K	phosphoinositide 3-kinase
RTK	receptor tyrosine kinase
RT-PCR	reverse transcription mRNA-PCRRA-level
TNF	tumor necrosis factor
WHO	World Health Organization
WM	Waldenstrom macroglobulinemia
WNT	wingless related integration site
